# Clinical Characteristics of SARS‐COV‐2 Omicron Variant in Acute Myeloid Leukemia and Acute Lymphocytic Leukemia Patients: A Multi‐Center Retrospective Study

**DOI:** 10.1002/cnr2.70146

**Published:** 2025-04-03

**Authors:** Lin Wang, Ruihua Mi, Lin Chen, Jia Liu, Haiping Yang, Meng Hu, Zhao Xiaoqiang, Yan Zhang, Xiaobing Xu, Bing Liu, Hongmian Zhao, Li Qianyu, Tao Liu, Chen Zhenzhu, Jinxiao Yao, Ying Yang, Xudong Wei

**Affiliations:** ^1^ The Affiliated Cancer Hospital of Zhengzhou University and Henan Cancer Hospital Zhengzhou China; ^2^ The First Affiliated Hospital, and College of Clinical Medicine of Henan University of Science and Technology Luoyang China; ^3^ Anyang District Hospital Anyang China; ^4^ Pingdingshan First People Hospital Pingdingshan China; ^5^ Huaihe Hospital of Henan University Kaifeng China; ^6^ Zhoukou Central Hospital Zhoukou China; ^7^ Nanyang Second People's Hospital Nanyang China

**Keywords:** acute leukemia (AL), acute lymphoblastic leukemia (ALL), acute myeloid leukemia (AML), COVID‐19, severe acute respiratory syndrome coronavirus 2 (SARS‐CoV‐2)

## Abstract

**Background:**

The death rate of hematological malignancies is high, and the death rate of patients with COVID‐19 infection is further increased. Although there have been expert consensus and relevant guidelines to introduce the recommendations of the guidelines for patients with hematological malignancies complicated with COVID‐19 infection, there is limited understanding of the clinical characteristics of Chinese patients with acute leukemia complicated with COVID‐19 infection.

**Aims:**

This study aimed to analyze the clinical manifestations, mortality, and determinants of viral shedding duration in Chinese AL patients infected with COVID‐19.

**Methods:**

We conducted a retrospective study of 100 AL patients with COVID‐19 infection in Henan Province, China, from December 1, 2022, to January 31, 2023. Data on demographics, leukemia subtype, symptoms, treatments (antibiotics/antivirals), and viral shedding duration were collected. Follow‐up was conducted over three months to assess mortality. Univariate and multivariate analyses were performed to identify risk factors.

**Results:**

The median age was 49.5 years (58% male, 42% female), with 76% having acute myeloid leukemia (AML) and 24% acute lymphoblastic leukemia (ALL). Most patients (86%) were asymptomatic. Antibiotics and antivirals were administered to 35% and 25% of patients, respectively. Severe cases and fatalities exhibited prolonged viral shedding. Neutropenic patients on antibiotics had significantly extended shedding duration, whereas antiviral therapy or delayed primary disease management shortened it. The overall mortality rate was 6%. Univariate analysis identified neutropenia as a key mortality risk factor, though multivariate analysis showed no significant associations.

**Conclusion:**

Early antiviral treatment may reduce viral shedding duration and potentially mitigate symptom severity and mortality in AL patients with COVID‐19. Neutropenia emerged as a critical factor influencing outcomes. These findings underscore the importance of tailored therapeutic strategies for this high‐risk population.

## 1 Introduction

Since 2019, SARS‐COV‐2 has spread globally, posing a significant threat to human health and life [1]. Due to the mutation in the spike protein on the virus's surface, SARS‐COV‐2 has evolved into various closely related mutants, including Alpha, Beta, Gamma, Delta, and Omicron [2, 3, 4, 5]. Although most patients with SARS‐CoV‐2 have mild symptoms, the infection spreads quickly and widely [6]. The mortality is significantly higher in patients with hematologic malignancies who are co‐infected with COVID‐19, especially in those with acute leukemia (AL) [7, 8]. AL, which includes acute myeloid leukemia (AML) and acute lymphoblastic leukemia (ALL), is a common type of hematological malignancy [9, 10]. Clinical management of AL in the context of SARS‐CoV‐2 infection has been addressed in expert consensus [11, 12]. However, our understanding of the clinical characteristics of AL patients with SARS‐COV‐2 infection remains limited. To better manage patients with AL infected with SARS‐COV‐2 and reduce associated mortality, we conducted a retrospective analysis of the general characteristics, treatment, and outcomes of AL patients with SARS‐CoV‐2 infection across multiple hospitals in Henan, China.

## 2 Methods

### 2.1 Study Design and Patients

This is a multicenter retrospective study, analyzing the clinical characteristics of AL patients afflicted with SARS‐COV‐2 in Henan Province, China, spanning from December 1, 2022, to January 31, 2023. The cohort of our patients originates from various hospitals. The inclusion criteria for AL patients were:

- Patients diagnosed with AL who have received leukemia‐related treatment within the past 5 years.
- Patients with a COVID‐19 nucleic acid test CT value of less than or equal to 36 [13].

The entirety of the patient's information is safeguarded, encompassing three distinct segments. The initial segment includes demographic details, such as age, gender, diagnostic categorization (AML/ALL), and physical status score. The second part consists of particulars concerning the AL, encompassing the most recent therapeutic regimen, the date of COVID‐19 diagnosis, and laboratory findings pre‐ and post‐infection (white blood cell count, neutrophil count/ratio, lymphocyte count/ratio, platelet count, c‐reactive protein, procalcitonin, creatinine, urea nitrogen, lactate dehydrogenase, D‐dimer, erythrocyte sedimentation rate). The third segment pertains to COVID‐19 specifics, including vaccination status against COVID‐19, the incidence of COVID‐19‐associated pneumonia, and the subsequent therapeutic measures post‐COVID‐19 infection.

### 2.2 Study Objectives

The primary aim was to analyze the gravity and lethality of AL patients infected with COVID‐19. The secondary research objectives encompassed: (1) An examination of the clinical attributes of AL patients contracting COVID‐19 infection (the repercussions on hematological indices, cardiac, hepatic, and renal functions). (2) An exploration of the therapeutic approach and the median duration of viral shedding which was delineated by the days during which COVID‐19 nucleic acid CT values remained less than or equal to 36. To accurately record the duration of viral shedding, we conduct throat swab nucleic acid tests every other day on patients diagnosed with COVID‐19 until the virus test results turn negative. Following the initial negative result, we perform tests for two consecutive days to confirm the stability of the outcome. The duration of viral shedding is defined as the period from the time of COVID‐19 diagnosis to the first instance of the virus turning negative.

### 2.3 Statistical Analysis

Categorical variables were characterized by their frequencies and percentages, whereas continuous variables were articulated as median, interquartile range (IQR), and absolute range. Univariate analysis was conducted on variables deemed to potentially influence the mortality of AL patients. Variables with *p* ≤ 0.1 were entered into multivariate Cox proportional hazard regression model. A *p* value ≤ 0.05 was considered statistically significant. SPSS v27.0 (SPSS, IBM Corp., Chicago, IL, United States) was employed for statistical analyses.

## 3 Results

From December 1, 2022, to January 31, 2023, a total of 906 patients were potentially at risk of contracting COVID‐19. We enrolled 115 patients with hematological malignancies complicated with COVID‐19 infection. After excluding 15 patients with MDS and lymphoma, our study encompassed 100 patients with AL. Among the 100 participants, there were 72 AML patients (72%), 24 ALL patients (24%), and 4 acute promyelocytic leukemia (APL) patients (4%). The general characteristics of patients with AL combined with COVID‐19 infection were shown in Table 1. The median age of all AL patients infected with COVID‐19 was 49.5 years old, including 58 male patients and 42 female patients. There were six newly diagnosed AL patients, 22 relapsed/refractory patients, and 72 patients in remission. Seventy‐two patients received chemotherapy within 1 month before or after COVID‐19 infection, and three patients received allogeneic hematopoietic stem cell transplantation within 1 month. After being infected with COVID‐19, 86 patients were asymptomatic or exhibited mild symptoms. Sixty‐six patients were hospitalized due to COVID‐19 infection and AL. After 3 months of follow‐up, six AL patients with COVID‐19 died. Among them, three perished due to active COVID‐19 infection, one due to the primary disease, and two due to a combination of COVID‐19 infection and primary disease.

**TABLE 1:** Clinical features of 100 AL patients combined with COVID‐19 infection: The table encompasses essential patient information, disease status, primary treatment modalities for the underlying condition, the severity of COVID‐19 infection, and the ultimate survival status of the patients.

	*n*
Age, median [range]	49.5 [2.9–83]
Sex	
Male	58
Female	42
Status AL at COVID‐19 diagnosis	
Newly diagnosed	6
Complete remission	72
Refractory/relapse	22
ECOG score	
< 2	68
≥ 2	32
Last treatment strategy before COVID‐19 diagnosis	
Treatment	93
Chemotherapy	76
Ongoing	27
Last month	45
Last 3 months	3
> 3 months	1
HSCT	17
Ongoing	1
Last month	2
Last 3 months	14
> 3 months	0
No treatment	7
Covid‐19 infection	
Asymptomatic	9
Mild infection	77
Severe infection	9
Critical infection	5
Stay during COVID‐19	
Admitted in hospital	66
Ordinary ward	60
ICU	6
Stay at home	34
Outcome	
Alive	94
Observation time, median days [range]	98 [77–161]
Dead	6
Observation time, median days [range]	20 [8–33]
Reason for death	
COVID‐19	3
Hematological malignancy	1
Covid‐19 & Hematological malignancy	2
Other reasons	0

**FIGURE 1:**
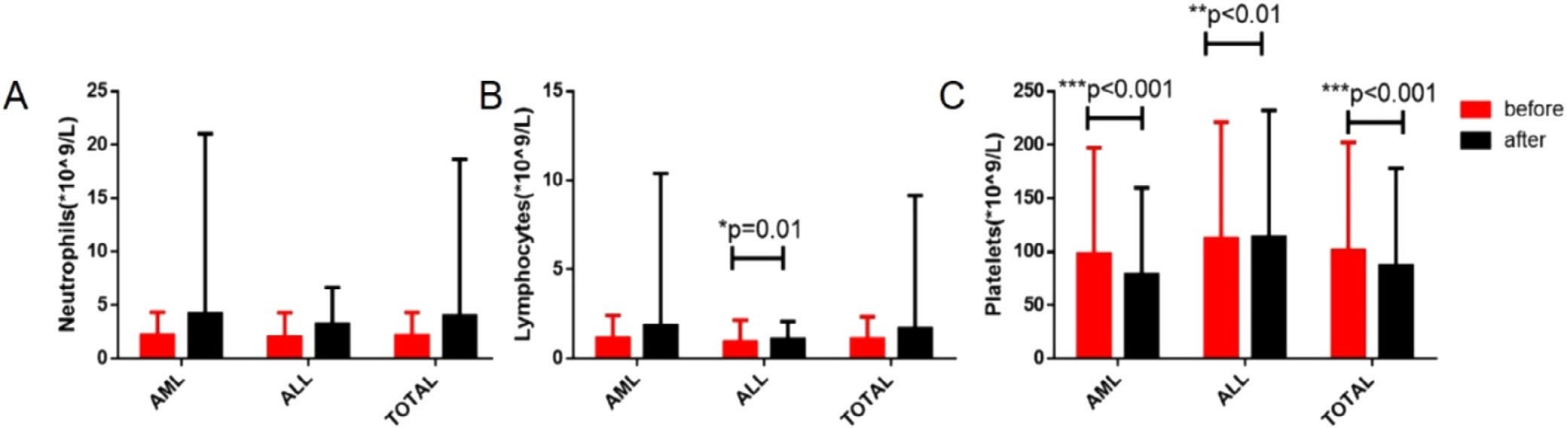
Neutrophil count, lymphocyte count, and platelet count in patients with acute leukemia before and after COVID‐19 infection (mean ± SEM, *p < 0.05, **p < 0.01, ***p < 0.001). (A) Neutrophil levels in patients with acute leukemia showed an increase post‐COVID‐19 infection as compared to pre‐infection levels; however, these changes did not achieve statistical significance. (B) Lymphocyte levels in patients with acute leukemia showed an elevation post‐COVID‐19 infection as compared with pre‐infection, with a notable *p* value of 0.01 observed specifically in ALL patients. (C) Platelet levels in patients with acute leukemia exhibited a reduction post‐COVID‐19 infection when compared with pre‐infection, and this decline was statistically significant (AML patients, *p* < 0.001, ALL patients, *p* < 0.01, Total patients, *p* < 0001).

At the onset of COVID‐19, 27 and 20 patients had neutrophil and lymphocyte counts below 0.5 × 10^9^/L and 0.2 × 10^9^/L, respectively, as shown in Table 2. Among AML patients, 21 and 13 had neutrophil and lymphocyte counts below 0.5 × 10^9^/L and 0.2 × 10^9^/L, respectively. Among ALL patients, six and seven patients had neutrophil and lymphocyte counts below 0.5 × 10^9^/L and 0.2 × 10^9^/L, respectively. When AL patients contracted COVID‐19 infection, they predominantly also suffered from neutropenia (with neutrophil count below 0.5 × 10^9^/L). The median duration of neutropenia was 8.5 days (all patients), 10 days (AML patients), and 6 days (ALL patients), respectively. Regarding treatment, 41% of patients did not require treatment related to COVID‐19. Among patients requiring treatment, 35 patients received antibiotics. And 25 patients received antiviral treatment, of which 4 received Paxlovid [14] and 16 received azvudine [15]. Forty‐five patients received glucocorticoid treatment, including 34 AML patients and 11 ALL patients. The median duration of viral shedding was 15 days in the whole sample, 15 days in AML patients, and 19.5 days in ALL patients. We also analyzed the impact of COVID‐19 infection on the neutrophil, lymphocyte, and platelet counts. The results showed that the neutrophil count of both AML and ALL patients increased after COVID‐19 infection, but there was no statistical significance. After COVID‐19 infection, the lymphocyte count of AL patients increased, and it was statistically significant in ALL patients (*p* = 0.01). COVID‐19 infection reduced the platelet count of AL patients, and this reduction was statistically significant in AML (*p* < 0.001) patients and ALL (*p* < 0.01) patients. (Figure 1).

**TABLE 2:** Laboratory features and treatment of 100 AL patients combined with COVID‐19 infection: The table encompasses the hematological parameters, clinical symptoms, treatments administered, and the duration of viral shedding COVID‐19 infection in patients with acute leukemia.

	AML	ALL	Total
Neutrophils ×10^9^/L (*n*)			
< 0.5	21	6	27
0.5–0.999	6	2	8
≥ 1	49	16	65
Lymphocytes ×10^9^/L (n)			
< 0.2	13	7	20
0.2–0.499	19	3	22
≥ 0.5	44	14	58
Days of neutrophils < 0.5 × 10^9^/L (median, range)	10 (1–32)	6 (0–23)	8.5 (0–32)
Days of temperature ≥ 38.5°C (median, range)	2 (0–20)	0.5 (0–10)	2 (0–20)
Treatment (*n*)			
Antibiotic	28	7	35
Antiviral			
Paxlovid	3	1	4
Azvudine	12	4	16
Others	5	0	5
Glucocorticoids	34	1	45
NE treatment	31	10	41
Days of CT ≤ 36 (median, range)	15 (4–38)	19.5 (3–33)	15 (3–38)

**FIGURE 2:**
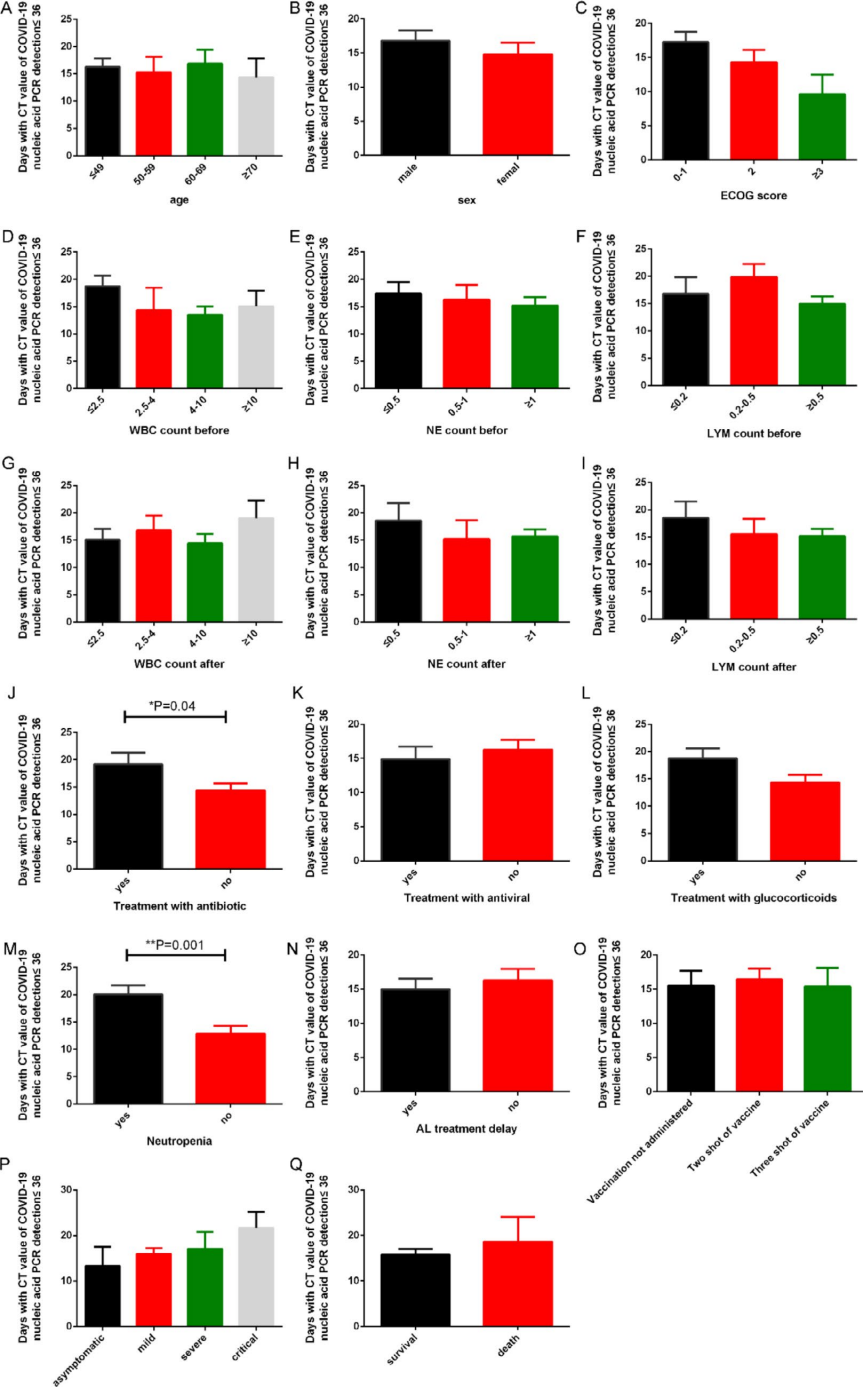
The impact of different factors on the duration days of viral shedding in AL patients. (mean ± SEM, *p < 0.05, **p < 0.01). Figures (A), (B), and (C) delineate the effects of age, gender, and ECOG scores on the duration of viral shedding, respectively. Figures (D), (E), and (F) elucidate the impacts of pre‐infection white blood cell count, neutrophil count, and lymphocyte count on the duration of viral shedding. Figures (G), (H), and (I) exhibit the influences of post‐infection white blood cell count, neutrophil count, and lymphocyte count on the duration of viral shedding. Figures (J), (K), and (L) depict the effects of antibiotic therapy, antiviral therapy, and glucocorticoids on the duration of viral shedding. Figure (M) indicates that the duration of viral shedding is notably prolonged in patients with concomitant neutropenia compared with those without, *p* = 0.001. Figure (N) illustrates the consequences of delayed treatment for acute leukemia on the duration of viral shedding. Figure (O) portrays the effects of varying COVID‐19 vaccination statuses on the duration of viral shedding. Figure (P) demonstrates the influence of the severity of COVID‐19 infection on the duration of viral shedding. Figure (Q) represents the effects of differing patient survival statuses on the duration of viral shedding.

We analyzed the duration of viral shedding following patient infection. Our results (Figure 2) revealed no significant disparities among AL patients across different age groups, genders, and ECOG scores. The varying levels of white blood cell, neutrophil, and lymphocyte counts prior to infection did not exhibit significant differences in the duration of viral shedding. However, the results showed that the lower neutrophil and lymphocyte counts after infection correlated with a longer duration of viral shedding, albeit without statistical significance. Patients complicated with neutropenia prior to the initiation of primary disease treatment exhibited a significantly longer duration days of viral shedding compared to those without neutropenia (20.08 days + 1.612 vs. 12.89 days + 1.393, *p* = 0.001). After COVID‐19 infection, delayed leukemia treatment (15.00 days ± 1.53 vs. 16.31 days ± 1.64) or antiviral therapy [14, 15] (14.87 days ± 1.87 vs. 16.30 days ± 1.39) shortened the duration, yet without a statistically significant difference. However, our results showed that the duration days of viral shedding in patients receiving antibiotic treatment was significantly longer than in those not receiving antibiotic treatment (19.15 days ± 2.18 vs. 14.39 days ± 1.27, *p* = 0.04). Although it was not statistically significant, the more severe the patient's symptoms, the longer the duration days of viral shedding. The severity grading of COVID‐19 was referenced according to previous literature reports [3]. The duration days of viral shedding in death cases was also significantly longer than in survivors (18.67 days ± 5.36 vs. 15.81 days ± 1.18, *p* > 0.05 for AL patients; 30.50 days ± 7.50 vs. 14.93 days ± 1.23, *p* = 0.01 for AML patients). In subgrouping analysis, the duration days of viral shedding between different subgroups of AML patients (Figure S1) was basically similar. Due to the limited sample size of ALL patients, subgroup analysis was not conducted.

Finally, we analyzed the factors influencing the mortality of AL patients infected with COVID‐19. The single‐factor analysis revealed that neutropenia in patients with COVID‐19, the severity of the infection, and the administration of glucocorticoids were associated with patient mortality. Nonetheless, the multivariate analysis yielded no statistically significant results (Table 3).

**TABLE 3:** Overall mortality predictors of death in AL patients with COVID‐19: Univariate and multivariate analyses were employed to discern factors affecting the mortality rate in acute leukemia patients with concurrent COVID‐19 infection. Univariate analysis revealed that neutropenia and the severity of COVID‐19 infection were risk factors influencing patient mortality; however, these factors did not achieve statistical significance in the multivariate analysis.

Overall mortality	AML, *p*		ALL, *p*		Total, *p*	
	Univariable	Multivariable	Univariable	Multivariable	Univariable	Multivariable
Sex	**0.034**.	0.99	0.495		0.292	
Age	0.654		0.210		0.494	
ECOG score	0.597		0.809		0.725	
WBC count before	**0.000**	0.984	0.894		0.75	
NE count before	0.319		0.768		0.973	
Lym count before	0.428		0.409		0.577	
PLT count before	0.44		0.957		0.774	
WBC count after	0.841		0.656		0.61	
NE count after	0.835		0.827		0.55	
Lym count after	0.866		0.58		0.672	
PLT count after	0.164		0.555		0.767	
Neutropenia	**0.032**	0.19	**0.008**	0.709	**0.002**	0.752
Treatment with antibiotic	0.36		0.898		0.389	
Treatment with antiviral	0.074		0.97		0.107	
Treatment with glucocorticoids	0.019	0.241	0.495		0.713	
Covid‐19 infection	**0.000**	0.98	**0.008**	0.899	**0.011**	0.961
AL treatment delay	0.289		0.212		0.197	

*Note:* A *p*‐value < 0.05 in univariate analysis was considered statistically significant and highlighted in bold.

## 4 Discussion

COVID‐19 poses a severe threat to the health of people worldwide. AL is characterized by its rapid onset and high mortality, necessitating early treatment. When AL patients are co‐infected with COVID‐19, the potential complications and the management of their primary disease pose significant challenges to clinicians. Therefore, through our research, we aim to gain a better understanding of the clinical characteristics and the progression of AL patients co‐infected with COVID‐19, thereby guiding clinicians in their diagnostic and therapeutic approaches.

Although the relevant expert consensus has been published regarding the primary clinical characteristics of AL patients infected with COVID‐19 and the selection of primary treatment scheme [11, 16], the clinical data of Chinese AL patients with COVID‐19 infection has not yet been evaluated [17, 18]. In this study, we analyzed the main clinical characteristics of Chinese patients with AL complicated with COVID‐19 infection and the impact on treatment strategies. The overall mortality of AL patients infected with COVID‐19 within 3 months was about 6%, significantly lower than the mortality rate previously reported internationally [8, 19]. This result is considered to be related to virus variability [20]. Furthermore, all patients underwent follow‐up 3 months after enrollment to ensure no patient was lost to follow‐up. The COVID‐19 variant of our patients was mainly Omicron [21, 22], and most patients exhibited mild symptoms and low mortality [17, 23]. However, the death of patients was mainly concentrated in the first month after infection. In line with previous studies, our results show that the neutrophil count of AL patients infected with COVID‐19 tends to increase [17]. Contrary to previous research findings, our data show an increasing trend in lymphocytes in AL patients after contracting COVID‐19 [24, 25], considering that most patients undergoing bone marrow suppression post‐chemotherapy. Following COVID‐19 infection, the platelet levels in patients with AL was significantly reduced, which was consistent with previous research results.

## 5 Conclusion

Our findings confirm for the first time that the duration of viral shedding in severe patients was significantly extended compared to those with mild symptoms, with a notably extended duration in death cases. The postponement of leukemia or antiviral treatment reduced the duration of viral shedding, albeit our results lacked statistical significance, given the small sample size. However, Neutropenia was associated with a prolonged duration.

In the assessment of mortality risk factors, univariate analysis revealed that neutropenia constituted a mortality risk factor in both AML and ALL patients, although the multivariate analysis results were not statistically significant. In contrast to previous research results, delayed chemotherapy exhibited no apparent correlation with patient survival.

Regarding treatment, the majority of AL patients with COVID‐19 complications experienced mild symptoms and did not require treatment. The administration of antibiotics, antiviral drugs, or glucocorticoids did not exert a significant impact on patient survival. The most known potential candidates against COVID‐19 includes Paxlovid, molnupiravir, and Azvudine [26, 27]. Paxlovid and molnupiravir have been shown to reduce hospitalization or death among patients with COVID‐19 who do not require hospitalization or supplemental oxygen both in clinical trials and in real‐world populations. Paxlovid was granted an Emergency Use Authorization for the treatment of mild to moderate coronavirus disease 2019 (COVID‐19), based on the interim analysis of the Evaluation of Protease Inhibition for COVID‐19 in High‐Risk Patients (EPIC‐HR) trial. Nucleoside analog 2′‐deoxy‐2′‐β‐fluoro‐4′‐azidocytidine, known as azvudine or FNC (MW, 286.22), is a prodrug that can be intracellularly converted into FNC triphosphate and inhibits viral RdRp. It has a broad‐spectrum activity against viruses, including HCV and EV71. FNC has been approved by China FDA for AIDS treatment on July 21, 2021 (XZXK‐2021‐214), showing efficacy in treating AIDS and good safety during the 48‐week oral treatment. Azvudine is the first Chinese oral anti‐COVID‐19 drug. Several clinical trials demonstrated that Azvudine could shorten the time for nucleic acid negative conversion in patients with COVID‐19 without adverse effects [28, 29, 30]. Due to the rapid spread of the COVID‐19 pandemic, Paxlovid and Molnupiravir have not yet been widely available in China, resulting in the majority of Chinese patients being unable to promptly access treatment with these two medications. Consequently, Azvudine has become the primary choice for treating COVID‐19 patients in China. Similar to previously published findings, our results indicate that antibiotic therapy does not confer benefit to patients. However, antiviral drugs or delaying the treatment of leukemia to a certain extent did shorten duration of viral shedding.

## 6 Limitations

We analyzed the data of Chinese patients with AL complicated with COVID‐19 infection. Additionally, we conducted an initial analysis of the factors influencing the duration of viral shedding. However, our cohort predominantly comprised adults with AML, limiting our understanding of the clinical characteristics of children with AL and COVID‐19 infection. Further research and long‐term follow‐up are needed to better understand the characteristics of AL patients with COVID‐19 infection.

## Author Contributions

Lin Wang, Ruihua Mi, Lin Chen designed research, performed research, analyzed data, wrote paper. Jia Liu designed research, contributed analytical tools. Haiping Yang, Meng Hu, Xiaoqiang, Zhao, Yan Zhang, Xiaobing Xu, Bing Liu, Hongmian Zhao, Li Qianyu, Tao Liu, Chen Zhenzhu, Jinxiao Yao, Ying Yang performed research, collected data, revised article. Xudong Wei designed research, analyzed data, revised article.

## Ethics Statement

The Human Investigation Committee of the Affiliated Cancer Hospital of Zhengzhou University approved this study.

## Consent

Informed consent was obtained from all individual participants included in the study. And all patients included in this study signed informed consent regarding publishing their data.

## Conflicts of Interest

The authors declare no conflicts of interest.

## Supporting information


**Figure S1.** The impact of different factors on days with CT value of COVID‐19 nucleic acid PCR detection ≤ 36 in AML patients (mean ± SEM, *p < 0.05, **p < 0.01).

## Data Availability

The data that support the findings of this study are available from the corresponding author upon reasonable request.
