# Supplementary material for: Clinical Characteristics of SARS‐COV‐2 Omicron Variant in Acute Myeloid Leukemia and Acute Lymphocytic Leukemia Patients: A Multi‐Center Retrospective Study
Source: Cancer Rep (Hoboken). 2025 Apr 3;8(4):e70146. doi: 10.1002/cnr2.70146 (PMC11965881; doi:10.1002/cnr2.70146)
Supplement: Supplementary file 1 — Figure S1. The impact of different factors on days with CT value of COVID‐19 nucleic acid PCR detection ≤ 36 in AML patients (mean ± SEM, *p < 0.05, **p < 0.01). [file CNR2-8-e70146-s001.docx]

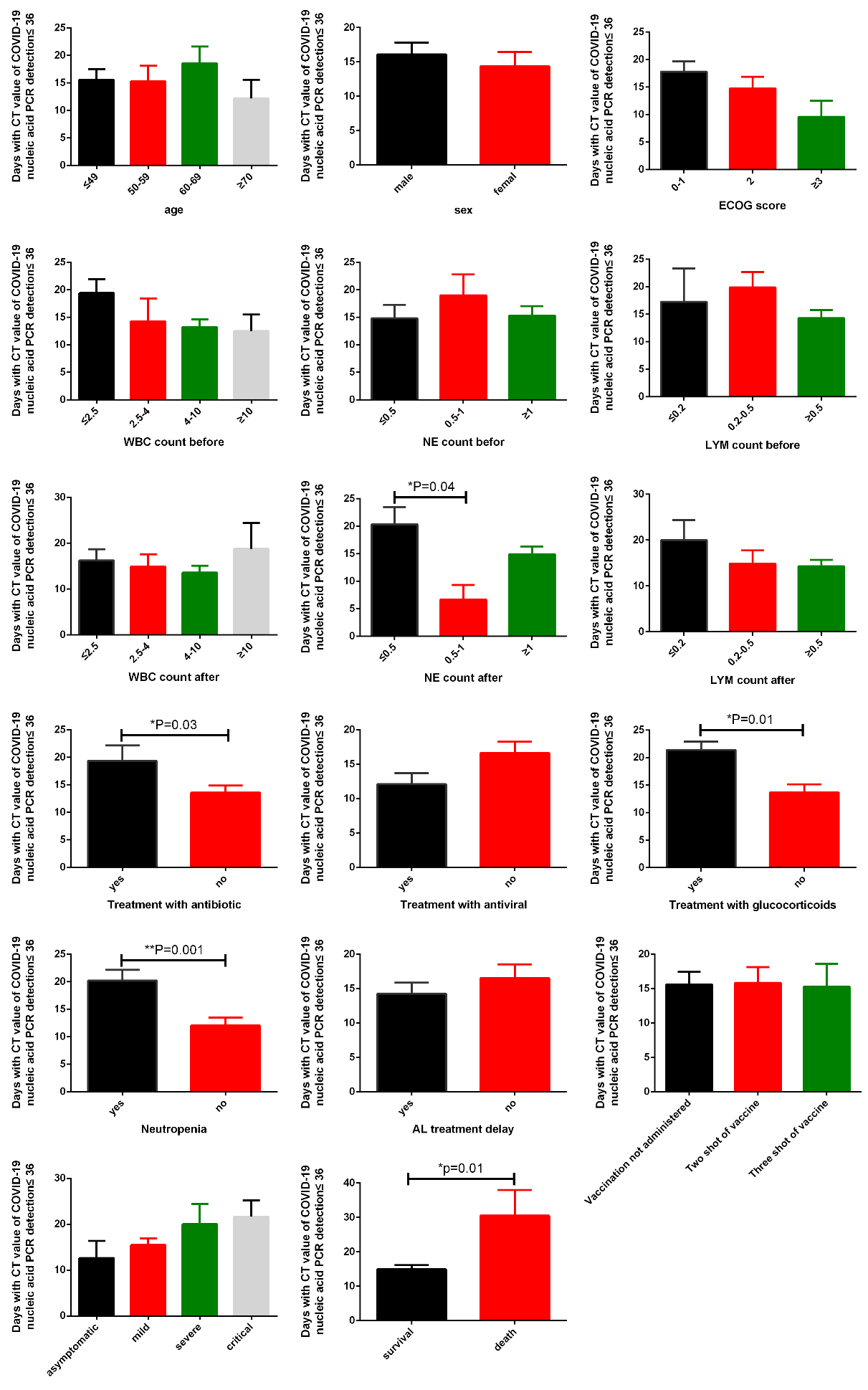


**Fig.1s** The impact of different factors on days with CT value of COVID-19 nucleic acid PCR detection≤36 in AML patients. (mean ± SEM,*P < 0.05, **P < 0.01).
